# The sibling effect on neurodevelopment of preschoolers under China’s newly relaxed child policy: A national retrospective cohort study

**DOI:** 10.3389/fpsyg.2022.988622

**Published:** 2022-12-06

**Authors:** Xiaotian Dai, Gareth Williams, Senran Lin, Charlie Baker, Meiqin Wu, Wenchong Du, Jing Hua

**Affiliations:** ^1^Shanghai Key Laboratory of Maternal Fetal Medicine, Shanghai First Maternity and Infant Hospital, School of Medicine, Tongji University, Shanghai, China; ^2^School of Social Science, Nottingham Trent University, Nottingham, United Kingdom; ^3^NTU Psychology, School of Social Science, Nottingham Trent University, Nottingham, United Kingdom

**Keywords:** China, sibling effect, neurodevelopment, family socioeconomic status, preschooler

## Abstract

**Introduction:**

The change in Chinese fertility policy brings new challenges and considerations for children’s health outcomes; however, very little is known about the interaction between siblings, family socioeconomic status (SES), and neurodevelopment in the Chinese preschool-age population. Therefore, this study aimed to develop a new explanatory pathway from sibling effect to early childhood development and explored the mediation effect of family SES in the pathway.

**Methods:**

From April 2018 to December 2019, we conducted a national retrospective cohort study in 551 cities in China, and a total of 115,915 preschool-aged children were selected for the final analysis. Children’s neurodevelopment, including Communication, Gross motor, Fine motor, Problem-solving, and Personal-social, was assessed with the Ages & Stages Questionnaires, Third Edition (ASQ-3). Hypothesis tests and multilevel regression models were used to assess the associations and their strength between sibling effect and neurodevelopmental delay. Pathway analysis was used to verify the mediation effect of SES.

**Results:**

The results showed that there were significant risk effects of a sibling on preschoolers’ overall neurodevelopment including communication, gross motor, fine motor, and problem-solving delay. The adjustment of family SES, however, brought a reversal of this association. The results of the mediation model illustrated a direct, protective effect of one-sibling status (βASQ-delay = −0.09; βASQ-scores = 0.07; *p* < 0.001), and an indirect, risk effect from one-sibling status through family SES to neurodevelopment outcomes (βASQ-delay =0.12; βASQ-scores = −0.12; *p* < 0.001). The total sibling effect was weakened but remained negative (βASQ-delay =0.03; βASQ-scores = −0.05; *p* < 0.001).

**Discussion:**

This study concluded that family SES mediated the negative effects of one sibling on early child development. To enhance the positive influence of sibling addition, we suggested providing more resources and instructions to the families with less educated and poorer employed parents under the coming multi-child era.

## Introduction

The effects of sibling presence on child development have been well examined; however, the results have been mixed. The resource dilution model suggested a negative association between family size and child development because more children in a family can lower the resources each child gets (Blake, 1981; Downey, 2001; Jaeger, 2009; Kalmijn and van de Werfhorst, 2016). Meanwhile, evidence has also highlighted that positive sibling interactions facilitate the neurodevelopment of children (Brody, 2004; Zhou et al., 2016; Du et al., 2020). For example, Zajonc and Markus’s Confluence Model theorized that a child’s development could benefit from a child-to-child teaching environment (Zajonc and Markus, 1975). More recently, an integrative model was proposed and emphasized the reciprocal and dynamic development of the sibling effect, which could be either negative or positive but constantly changing with age (Hou et al., 2020). Still, other studies find no relationship between child quantity and quality (Pennington and Harpending, 1988; Draper and Hames, 2000; Jamison et al., 2002; Tada et al., 2002; Hesketh et al., 2003).

Previous studies may be inconsistent for different reasons. On the one hand, naturally occurring teaching and caregiving experiences between older and younger siblings can benefit cognitive, language, and psychosocial development in both children (Brody, 2004). Also, parents’ experiences with older children can lead to effective child-rearing strategies for the subsequent child (Whiteman and Buchanan, 2002). On the other hand, more children in a family can also dilute available parental resources per child (Wu, 2015). As suggested by the resource dilution model, there are universal signs of the trade-off between child development and family size, but the trade-off may only be prominent when the family resources are limited (Gillespie et al., 2008), or the family socioeconomic status (SES) is low.

The complicacy of family SES makes it a unique context for children’s development. It was widely reported that family SES disadvantage could negatively affect neurodevelopment from infancy to school-aged children (Bradley and Corwyn, 2002; Tong et al., 2007; Schady, 2011; Ronfani et al., 2015). Some researchers suspected that the actual causal relation between sibling effect and child development may involve family SES as a confounder rather than a mediating factor (Steelman, 1985). That is, parents in low SES families are more likely to have more children, and SES influence is not adequately adjusted for a statistical effect of a sibling on development. However, in modern developed countries, more children per family do not necessarily mean more labor force but rather more parental care responsibilities, and parents’ fertility decisions may be affected by family SES in a different way (Tong and Gong, 2020). In that case, the sibling effect on child development might be more prominent because the influence of family SES has been mediated. Therefore, the role of the family SES in the association of the presence of siblings with child development needs to be further clarified.

However, most of the work in this field has been based on families from Western countries. Due to China’s 35 years of one-child restrictions, limited studies have focused on the association between sibling status and early childhood development. Moreover, the one-child policy has significantly affected China’s social and family structure, people’s life patterns, and parenting styles (Cheng, 2005; Ding and Hesketh, 2006; Cheng, 2013). The persistent son preference in the Chinese culture and sex-selective birth (Hua et al., 2016; Huang et al., 2016; Liu et al., 2017) may pose additional challenges to clarify the sibling effect given that unobserved factors may jointly determine the decision to have a second child and the potential unequally allocated resources among children.

There are several reasons why it is particularly important to investigate the sibling effect on child development in the context of relaxing the child control policy in China. First, the low-fertility rate in China (both under the one-and two-child policy) has been mainly driven by government regulations instead of personal decisions, and the parents who decided to have a second child under a relaxed child policy may reflect different sociodemographic characteristics distinct to those in other countries (Hua et al., 2016). Second, because of the longstanding one-child culture in China before 2016, parents who decided to have a second child under the newly relaxed child policy in China may be the ones who are more eager or ready for a second child (Chen, 2020). If only those parents who can maintain the level of family resources in the existing child decide to have another child, then a second child may not affect the access to family resources because of self-selection.

To our knowledge, there is no national population-based study after China relaxed its child control policy in 2016. The current study, therefore, had two aims. First, with a national representative sample, we were aiming to address the understudied question of whether the presence of a sibling is associated with neurodevelopment in Chinese preschoolers aged 3–5 years old under China’s newly relaxed two-child policy. Second, we would like to examine the specific role of family SES in the association between sibling status and neurodevelopment. We hypothesized that the presence of a sibling had a negative effect on neurodevelopment, while the family SES could mediate the negative effect in the pathway.

## Materials and methods

### Participants

**Figure 1:**
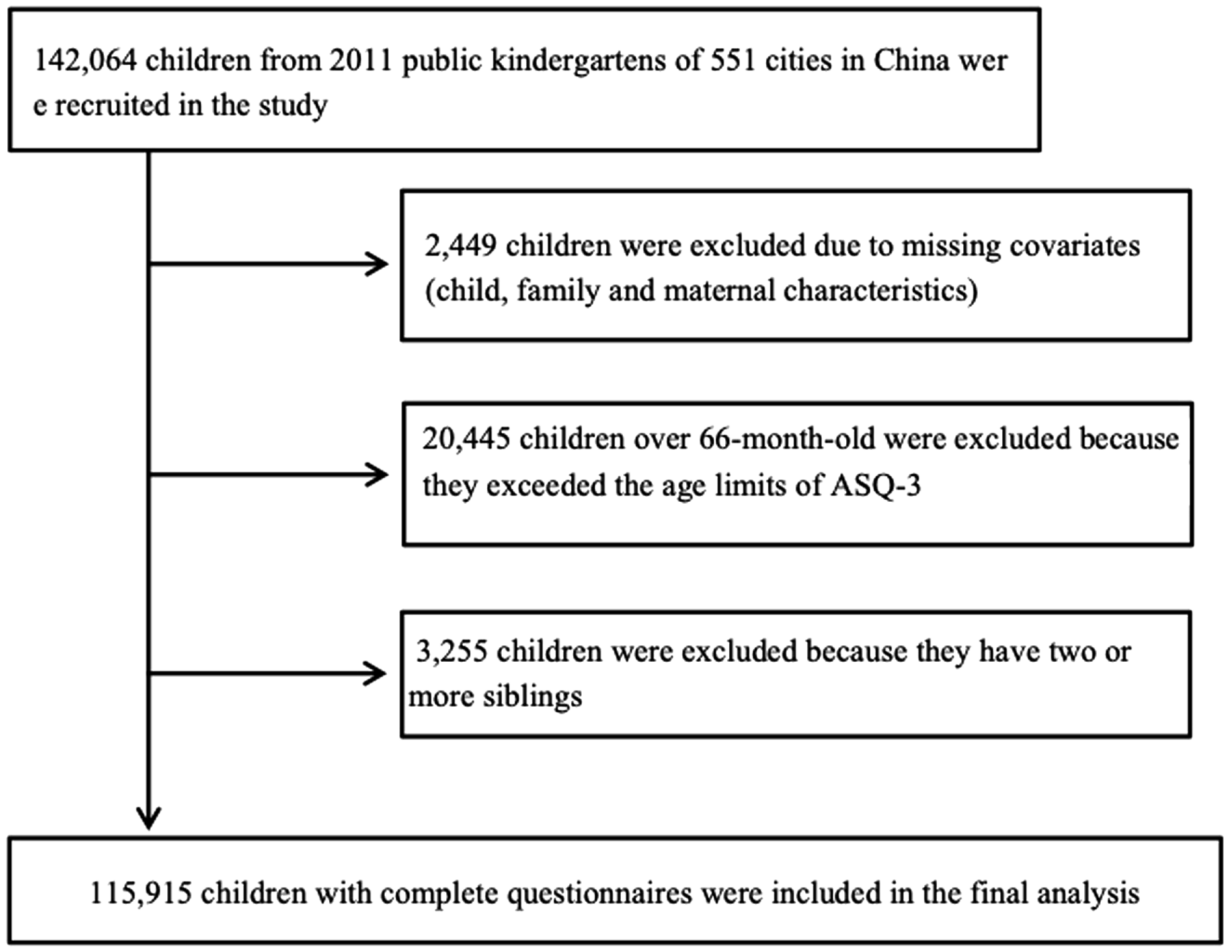
Flowchart of the study population.

A national retrospective cohort study was conducted in China from April 2018 to December 2019. The recruitment followed a cluster sampling plan that covered all administrative districts in mainland China, including 4 direct-controlled municipalities, 5 autonomous regions, and 22 provinces to ensure a representative study sample of the Chinese population. A total of 142,064 preschoolers from 2011 public kindergartens in 551 cities of China were enrolled using area, gender, age, and SES as stratification variables. Children with normal intelligence and no physical disabilities, who had parents with sufficient proficiency in completing the online questionnaire, were eligible for the study. After excluding the children aged over 66 months, with two or more siblings,^1^ or with missing values of their investigation, 115,915 children were selected for the final analysis (see Figure 1).

The study protocol was approved by the Ethics Committee of Shanghai First Maternity and Infant Hospital (KS18156). For ethical concerns and information safety, all the data collected were kept confidential and only accessible to the researchers.

### Procedure

We developed an electronic questionnaire system to collect and manage large data. All selected cities have their independent Quick Response (QR) codes to access the questionnaires. Local kindergartens were informed to complete the investigation and supervised by the government-supported maternity and children’s health care center in each city. Class teachers were responsible for distributing the notification and QR code to the parents to fill out the questionnaire. Researchers’ contact information was provided if parents had queries about the study or about how to answer the questionnaires.

### Outcomes

Children’s neurodevelopment was assessed using the Ages & Stages Questionnaires, Third Edition (ASQ-3), a parent-completed questionnaire. It is a developmental screening tool targeted at children between the ages of 1 to 66 months across five domains: Communication, Gross motor, Fine motor, Problem-solving, and Personal-social. Six items with three options (YES = 10, SOMETIMES = 5, NOT YET = 0) in each area pinpoint the developmental progress of children. The ASQ scores are added and compared with cutoffs resulting in two kinds of outputs: 0 = typical development (scores higher than 2.0 SD below the mean), 1 = suspected developmental delay (scores ≤ 2.0 SD below mean). Both continuous and binary outcomes were analyzed. In the study, we use the Chinese language version which has reported a reliability coefficient of 0.8 (*p* < 0.001; Wei et al., 2015).

### Predictor and mediators

Sibling number was reported by parents. Children with more than two siblings were not included in the current study. The predictor was binary: 0 = no sibling, 1 = one sibling.

Five indicators were included in the current study for family SES to describe a more thorough description of the family social-economic status: higher education of mother, higher education of father, mother’s occupation, father’s occupation, and family annual per-capita income (RMB). Individuals reported their education by selecting one of six options ranging from having no formal education to having a master or PhD degree. Parents with a college degree or above were defined as being higher educated. Occupation status was divided into the two categories of employed or unemployed. The self-reported family annual per-capita income (RMB) was compared with the national average of the year before the survey time.

### Covariates

We included the child, family, and maternal health characteristics as potential confounders. Most of these variables were categorical; Body mass index (BMI) is an indicator of nutritional condition which is based on height and weight (BMI = weight(kg)/height(m)) according to the world health organization (WHO) BMI classification (WHO Expert Consultation, 2004); maternal age at birth was classified into three age bands: ‘< 30’, ‘30–34’ and ‘> 34’ per the literature (Teng et al., 2020); and gestational age (weeks) was divided into three groups: ‘< 37’, ‘37–41’ and ‘> 41’.

### Data analysis

All statistical analysis was performed using R Statistical Software (v4.1.2; R Core Team, 2021). The children’s neurodevelopment outcomes (ASQ total scores, Communication, Gross motor, Fine motor, Problem-solving, and Personal-social) and family SES were compared, respectively, between single-child and one-sibling status by *t*-test and Pearson’s Chi-square test. We conducted a simple mixed model on a kindergarten level and individual level utilizing a random intercept for calculating the intraclass correlation coefficient (ICC). Then multilevel linear and logistic regression models were used to assess the associations and their strength between China’s two-child family status and children’s neurodevelopmental delay in each domain of ASQ-3 when the SES were considered or not considered.

We used a mediation model with a structural equation modeling (SEM) approach to estimate the direct and indirect effects of a sibling. The ASQ scores and family SES were modeled as latent variables. Path models were estimated using weighted least squares (WLS) for binary outcomes and maximum likelihood (ML) for continuous outcomes. We used backward stepwise selection and model fit statistics (comparative fit index [CFI], Tucker-Lewis index [TLI], root-mean-square error of approximation [RMSEA], standardized root mean square residual [SRMR], Akaike Information Criteria [AIC], and Bayesian Information Criteria [BIC]) to determine best-fitting models.

## Results

### Sample characteristics

In the sample of 115,915 preschoolers, 50,613 (43.66%) were from two-child families and 65,302 (56.34%) were from one-child families. Over half of the total population (52.40%), single-child family population (53.40%), and two-child family population (51.10%) were male. There were significant differences between single-child and two-child families in the distribution of BMI, gender, gestational age, and maternal age at birth (*p* < 0.001). Age and delivery mode, however, were similar between children in two kinds of family size statuses (*p* > 0.05, see Table 1).

**Table 1:** The general characteristics and ASQ scores of preschool students (*N* = 115,915).

	Total	Single-child status	One-sibling status	*p*
**General characteristics**				
Age; Mean (SD)^a^	4.2110(0.75)	4.2076(0.75)	4.2153 (0.74)	0.079
				
BMI; Mean (SD)^a^	15.6704(2.11)	15.6376(2.11)	15.7126(2.12)	<0.001
				
Gender *N* (%)^b^				
Male	60,735(52.40)	34,873(53.40)	25,862(51.10)	<0.001
Female	55,180(47.60)	30,429(46.60)	24,751(48.90)	
Gestational age (weeks) *N* (%)^b^				
<37	10,091(8.71)	5,337(8.17)	4,754(9.39)	<0.001
37–41	101,660(87.70)	57,521(88.08)	44,139(87.21)	
>41	4,164(3.59)	2,444(3.74)	1720(3.40)	
Maternal age at birth *N* (%)^b^				
<30	87,460(75.45)	54,803(83.92)	32,657(64.52)	<0.001
30–34	21,108(18.21)	8,728(13.37)	12,380(24.46)	
≥35	7,347(6.34)	1771(2.71)	5,576(11.02)	
Delivery mode *N*(%)^b^				
Vaginal birth	60,530(52.22)	33,984(52.04)	26,546(52.45)	0.168
Cesarean section	55,385(47.78)	31,318(47.96)	24,067(47.55)	
				
**ASQ; Mean (SD)**^a^				
Total score	259.93(44.15)	261.20(43.19)	258.29(45.31)	<0.001
Communication	53.98(9.83)	54.31(9.56)	53.55(10.15)	<0.001
Gross motor	49.96(12.01)	50.16(11.89)	49.71(12.21)	<0.001
Fine motor	48.08(13.06)	48.37(12.94)	47.69(13.20)	<0.001
Problem-solving	53.68(1.00)	54.10(9.70)	53.15(10.34)	<0.001
Personal-social	54.23(90.22)	54.26(8.91)	54.18(9.16)	0.14

^a^Independent-sample *t*-test; ^b^Pearson’s Chi-square.

### Neurodevelopment outcomes

Table 1 also shows the difference in ASQ-3 scores between preschoolers with or without a sibling. Children with one sibling demonstrated lower outcomes in total score (258.29 ± 45.31), communication (53.55 ± 10.15), gross motor (49.71 ± 12.21), fine motor (47.69 ± 13.20), and problem-solving (53.15 ± 10.34) compared to the single child (*p* < 0.001). The personal-social score, however, was similar between the two statuses (*p* = 0.140). The associations between children’s neurodevelopment outcomes and their families’ SES are presented in Table 2.

**Table 2:** The mean scores of ASQ-3 in children with different socioeconomic characteristics (*N* = 115,915).

	Total score	Communication	Gross motor	Fine motor	Problem-solving	Personal-social
	*M*(SD)***	*M*(SD)***	*M*(SD)***	*M*(SD)***	*M*(SD)***	*M*(SD)***
**Higher education of mother**						
No	248.02(47.66)	51.70(10.87)	47.00(13.10)	44.88(13.85)	51.37(11.11)	53.05(9.80)
Yes	269.07(38.86)	55.72(8.56)	52.23(10.57)	50.53(11.85)	55.45(8.64)	55.13(8.26)
**Higher education of father**						
No	249.21(47.38)	51.95(10.76)	47.33(12.98)	45.21(13.78)	51.59(11.02)	53.13(9.74)
Yes	268.60(39.27)	55.62(8.67)	52.09(10.71)	50.40(11.95)	55.37(8.73)	55.12(8.29)
**Mother’s occupation**						
Unemployed	252.42(46.89)	52.39(10.66)	48.21(12.65)	46.13(13.68)	52.30(10.76)	53.38(9.69)
Employed	261.38(43.45)	54.28(9.63)	50.30(11.86)	48.45(12.90)	53.95(9.82)	54.39(8.88)
**Father’s occupation**						
Unemployed	245.60(55.91)	50.74(12.61)	46.52(14.32)	45.08(14.76)	50.85(12.41)	52.42(11.25)
Employed	260.32(43.72)	54.07(9.73)	50.06(11.93)	48.16(13.00)	53.76(9.91)	54.28(8.95)
**Family annual per-capita income (RMB)**						
Below	254.37(46.06)	52.87(10.34)	48.53(12.56)	46.61(13.59)	52.71(10.48)	53.64(9.37)
Above or equal to	261.77(43.34)	54.34(9.63)	50.44(11.79)	48.56(12.84)	54.01(9.81)	54.42(8.89)

***p < 0.001.

Socioeconomic status was significantly associated with preschoolers’ ASQ total score, communication, gross motor, fine motor, problem-solving, and personal-social development (*p* < 0.001). All mean scores significantly increased when either mother or father had higher education. Compared to the father, the mother with a bachelor’s degree or above brought more increments in children’s total scores from 248.02 to 269.07, communication from 51.70 to 55.72, gross motor from 47.00 to 52.23, fine motor from 44.88 to 50.53, problem-solving from 51.37 to 55.45, and personal-social from 53.05 to 55.13. Having an employed parent contributed to improving children’s neurodevelopment, while children with an unemployed father have lower scores in every ASQ-3 area than those with an unemployed mother. Differences between children from families with different incomes also exist. Children whose family annual per-capita income (RMB) is equal to or above the national average have significantly higher scores in total (261.77 ± 43.34), communication (54.34 ± 9.63), gross motor (50.44 ± 11.79), fine motor (48.56 ± 12.84), problem-solving (54.01 ± 9.81), and personal-social (54.42 ± 8.89).

### One–sibling effects

There were no gender or age differences in the association between sibling effect and neurodevelopment (*p* > 0.05). The crude and SES-adjusted coefficients of the predictor in our multilevel linear regression models are shown in Table 3. In the crude model, one-sibling status was significantly associated with every domain of ASQ. There were negative sibling effects on total score (*β* = −1.31, *p* < 0.001), communication (*β* = −0.50, *p* < 0.001), gross motor (*β* = −0.15, *p* < 0.05), fine motor (*β* = −0.33, *p* < 0.001), and problem-solving (*β* = −0.63, *p* < 0.001) outcomes. However, one-sibling status benefited children’s personal-social performance (*β* = 0.17, *p* < 0.01). After family SES was adjusted, the positive association between sibling effect and personal-social was enhanced (*β* = 0.48, *p* < 0.001). Furthermore, the adjustment of family SES converted the sibling effect on ASQ total score (*β* = 1.43, *p* < 0.001), gross motor (*β* = 0.54, *p* < 0.001), and fine motor (*β* = 0.45, *p* < 0.001) from negative to positive. The correlations between one-sibling status and communication or problem-solving outcomes, however, lost their significance (*p* > 0.05).

**Table 3:** The association between sibling effect and ASQ-3 scores in preschoolers (*n* = 115,915).

	Single-child status	One-sibling status^a^	
		Crude β^b^ (95% CI)	Adjusted β^c^ (95% CI)
ASQ total score	Reference	−1.31*** (−1.58, −1.04)	1.43*** (1.17, 1.70)
Communication	Reference	−0.50*** (−0.56, −0.44)	0.04 (−0.02, 0.10)
Gross motor	Reference	−0.15* (−0.23, −0.08)	0.54*** (0.47, 0.61)
Fine motor	Reference	−0.33*** (−0.41, −0.25)	0.45*** (0.37, 0.53)
Problem-solving	Reference	−0.63*** (−0.69, −0.57)	−0.11 (−0.17, −0.05)
Personal-social	Reference	0.17** (0.11, 0.22)	0.48*** (0.42, 0.53)

^a^*p < 0.05, **p < 0.01, ***p < 0.001; ^b^Adjusting for children’s general characteristics; ^c^Adjusted for children’s general characteristics and socio-economical characteristics.

**Figure 2:**
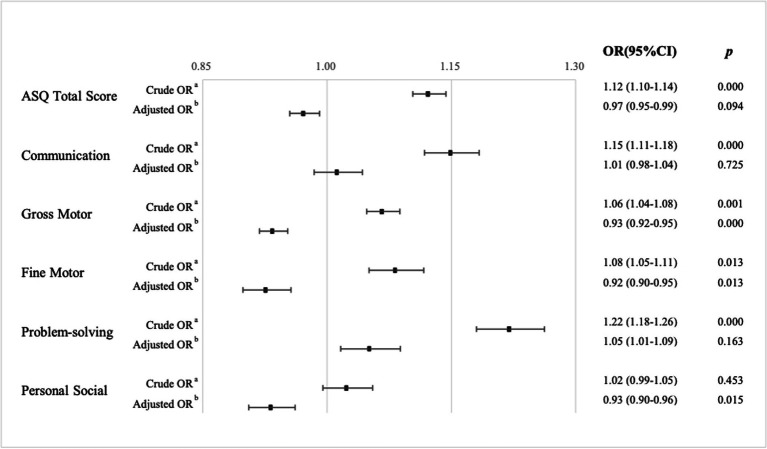
The association between sibling effect and risk of neurodevelopmental delays in preschoolers. ^a^Adjusted for children’s general characteristics; ^b^Adjusted for children’s general characteristics and socio-economical characteristics.

Figure 2 displays the crude and adjusted odds of ASQ-3 delay, given single-child status compared to one-sibling status. Regardless of any SES factor, the crude odds ratios showed significant risk effects of a sibling on children’s ASQ total score (OR = 1.12; 95%CI: 1.10, 1.14; *p* < 0.001), communication (OR = 1.15; 95% CI: 1.11, 1.18; *p* < 0.001), gross motor (OR = 1.06, 95% CI: 1.04, 1.08, *p* < 0.01), fine motor (OR = 1.08; 95% CI: 1.05, 1.11; *p* < 0.05), and problem-solving (OR = 1.22, 95% CI: 1.18, 1.26, *p* < 0.001) delay. At the same time, we observed no association between one-sibling status and personal-social development in the crude model (*p* > 0.05). Once family SES was adjusted, the risk effect of a sibling on ASQ total score, communication, and problem-solving performance was missing (*p* > 0.05). By contrast, the sibling effect on motor development was inverse. Both odds of gross motor (Adjusted OR = 0.93, 95% CI: 0.92, 0.95, *p* < 0.001) and fine motor (Adjusted OR = 0.92, 95%CI: 0.90, 0.95, *p* < 0.05) decreased to less than 1 and remained significant in the adjusted model. The protective effect of one-sibling status was present in the personal-social area (Adjusted OR = 0.93, 95% CI: 0.90, 0.96, *p* < 0.05).

### The mediation effect of family SES

**Figure 3:**
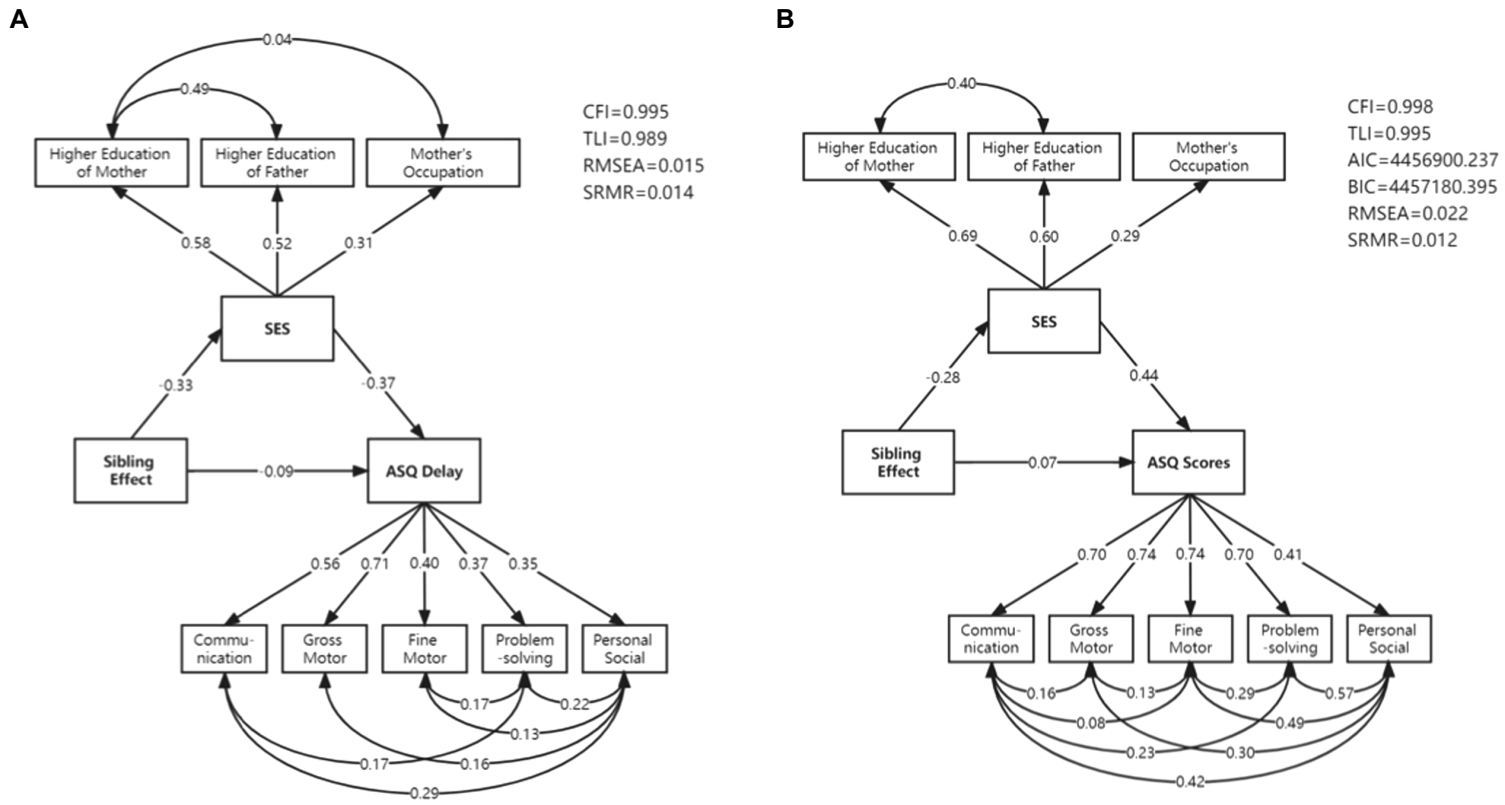
The mediation model diagrams: **(A)** binary outcomes (ASQ delay); **(B)** continuous outcomes (ASQ scores).

Mediation analysis was conducted for binary outcomes (ASQ delay) and continuous outcomes (ASQ scores) separately. The path diagrams of the best-fitting models are included in Figures 3A,B. All the standardized regression coefficients shown in Figure 3 were statistically significant (*p* < 0.001). Family SES was modeled as a latent variable by only three factors: higher education of the mother, higher education of the father, and the mother’s occupation, according to the backward elimination process and model fit statistics. In the binary outcomes model, the CFI, TLI, RMSEA, and SRMR were 0.995, 0.989, 0.015, and 0.014, respectively (AIC or BIC was not reported as they were not available for WLS estimation). Correspondingly, the CFI, TLI, AIC, BIC, RMSEA, and SRMR of the continuous outcomes model were 0.998, 0.995, 4456900.237, 4457180.395, 0.022, and 0.012. The results of the two models were similar, which illustrated a direct, protective effect of one-sibling status (β_ASQ-delay_ = −0.09; β_ASQ-scores_ = 0.07; *p* < 0.001), and an indirect, risk effect from one-sibling status through family SES to ASQ outcomes (β_ASQ-delay_ = 0.12; β_ASQ-scores_ = −0.12; *p* < 0.001). The total sibling effect on children’s neurodevelopment was weakened but remained negative (β_ASQ-delay_ = 0.03; β_ASQ-scores_ = −0.05; *p* < 0.001).

## Discussion

To our knowledge, this was one of the largest national retrospective cohort studies in China to explore various health outcomes in children (Zwicker, 2021). In this analysis, we found the presence of a sibling resulted in high risks of multiple neurodevelopmental delays. But the negative sibling effect was reversed when family SES was controlled. By path analysis, the mediation effect of SES on the paths from one-sibling status to children’s neurodevelopment was evident in our models. The sibling effect was directly protective against worsening neurodevelopment but indirectly led to suspected neurodevelopmental disorders through SES. Our results suggested that the risk of sibling effect on neurodevelopment existed when family resources were too limited to be shared.

We observed a negative sibling effect on neurodevelopment among Chinese preschoolers. The presence of a sibling increased the prevalence of suspected communication, gross motor, fine motor, and problem-solving delays. Our finding keeps in line with the prior national population-based studies set in China (Li et al., 2008; Wu, 2015). Notably, however, the adjustment of family SES brought a reversal of this association, which indicated the risk was conditional. Our results supported the hypothesis that considers sibling a spurious effect. That is, the negative effect of divided resources of siblings would reveal when the disadvantage of parents’ lower SES could not be fully modified. The findings provide a probable explanation for the inconsistent outcomes from previous studies in rural (Zhong et al., 2019) and urban (Wu et al., 2022) China.

Our results emphasized the importance of general parenting resources in early development, which is consistent with the Resource Dilution Model (Blake, 1981; Downey, 2001; Kalmijn and van de Werfhorst, 2016). Furthermore, we observed that “parenting resources” could be well predicted by the family SES (education, vocation, and income status). Both parental education and occupation were associated with better parenting and home resources, which is highly consequential for neurodevelopment, especially during the earliest years of children’s lives. A parent with a higher educational level is better able to adapt parenting abilities and maximally utilize family or community resources (DeGarmo et al., 1999). Ultimately, young children’s development is highly dependent upon parental inputs (Shonkoff et al., 2012). Following our study results, we deem parents’ education, vocation, and income as the source of these inputs which can be partitioned by the addition of a sibling. Crucially, if the overall pool of resources is sufficient for the children’s wellbeing, there will be no limitation, but rather benefit for two-child families. However, for families in low SES situations, the extra sibling might occupy the parenting resources and a negative sibling effect could occur.

Family SES functioned as a mediator linking sibling effect to neurodevelopment. Path analysis of psychological processes helped us break down the association into components to better understand the possible causal mechanisms (Shrout and Bolger, 2002). We determined that the presence of a sibling had a negative total effect on neurodevelopment which consequently explained the apparent correlation in crude models. Numerous researchers have reported lower SES is inversely associated with early development (Ursache and Noble, 2016; Vukojević et al., 2017; Takeuchi et al., 2021). This study adds support to the few existing studies on the relation between family SES and the number of children in China, providing evidence that sibling addition was related to lower SES. For model optimization, we removed the father’s occupation and family incomes from the models. Parental education, specifically the mother’s higher education level brought the most disparity in all five domains among preschoolers. Our results theoretically support the previous national research finding that the negative effect of an additional sibling is mainly driven by the family with a low educated mother (Chen, 2020). In Chinese family values, mothers typically perform the child-rearing role while fathers tend to be responsible for making money. More educated mothers were more likely to introduce healthy rearing behaviors while working mothers provide families with better resources.

Our sample was generally born around the year 2016 and raised in the two-child policy era, which gave us a comprehensive review of the unique Chinese fertility influence. According to a survey released by the All-China Women’s Federation (ACWF, 2017), the two-child policy has brought severe pressure to the healthcare and education systems, and the competition for the limited resources may lead to an even worse situation for lower SES families. The negative association between the sibling effect and early development becomes relatively stronger especially when there is little social support for children (Park, 2008). Therefore, providing more external resources to preschoolers in lower SES families is an urgent concern, especially social support on health education and surveillance of family planning and children’s development. Decision-makers might consider a targeted preschool childcare service as complementary stimulation for cognitive development outside the home environment during early childhood.

The main limitation of our study is the lack of adjustment for some variables of the parenting style with Chinese characteristics, i.e., children raised by grandparents, and some variables potentially associated with the neurodevelopment outcomes, i.e., birth order. In addition, this result was based on a cross-sectional design that could not completely control for some potential effects shared between siblings over time. Therefore, further analysis of the longitudinal development data would better provide a casual prediction.

## Conclusion

This study was conducted to explore the interaction between siblings, family socioeconomic status, and neurodevelopment in the Chinese preschool-age population. The results confirm that family SES mediated the effects of one sibling on the neurodevelopment of preschoolers under China’s newly relaxed child policy. Our nationally representative results have significant theoretical implications for both healthcare workers and decision-makers.

It was the first time to use SES to build up a cross-bridge between sibling effect and children’s neurodevelopment outcomes. In particular, children with a sibling would be at risk of multiple neurodevelopmental delays if they belonged to low SES families. As the change in Chinese fertility policy will bring new challenges and considerations for children’s health outcomes, we suggest providing more resources and instructions to families with less educated and poorer employed parents under the coming multi-child era.

## Data availability statement

The raw data supporting the conclusions of this article will be made available by the authors, without undue reservation.

## Ethics statement

The studies involving human participants were reviewed and approved by the Ethics Committee of Shanghai First Maternity and Infant Hospital (KS18156). Written informed consent to participate in this study was provided by the participants’ legal guardian/next of kin.

## Author contributions

XD was responsible for the data analysis and paper writing. GW edited and revised the paper. SL contributed to the data collection and management. CB and MW were responsible for proofreading. WD designed the study and oversaw the writing quality. JH made the statistical strategy and took charge of the whole project. All authors contributed to the article and approved the submitted version.

## Funding

The study was financially supported by the National Natural Science Foundation of China (81673179), the Science and Technology Commission of Shanghai Municipality (19140903100), Shanghai Municipal Health Commission (2020YJZX0213), Shanghai Pudong New Area Health Commission, and Clinical Research Plan of Shanghai Hospital Development Center (SHDC2020CR1047B-003).

## Conflict of interest

The authors declare that the research was conducted in the absence of any commercial or financial relationships that could be construed as a potential conflict of interest.

## Publisher’s note

All claims expressed in this article are solely those of the authors and do not necessarily represent those of their affiliated organizations, or those of the publisher, the editors and the reviewers. Any product that may be evaluated in this article, or claim that may be made by its manufacturer, is not guaranteed or endorsed by the publisher.

## Footnotes

^1^Before May. 2019, the two-child limit was enforced on urban residents. But in rural area, or in minorities, or for some special medical reasons, couples may have three or more children.

## References

[ref1] ACWF. (2017). *Two-child Policy’s Impact on Family Education, in Women of China English Monthly*. Beijing, China: Women’s Foreign Language Pulications of China, p. 68–69.

[ref2] Blake J. (1981). Family-size and the quality of children. Demography 18, 421–442. doi: 10.2307/2060941, PMID: 7308532

[ref3] Bradley R. H. Corwyn R. F. (2002). Socioeconomic status and child development. Annu. Rev. Psychol. 53, 371–399. doi: 10.1146/annurev.psych.53.100901.135233, PMID: 11752490

[ref4] Brody G. H. (2004). Siblings’ direct and indirect contributions to child development. Curr. Dir. Psychol. Sci. 13, 124–126. doi: 10.1111/j.0963-7214.2004.00289.x, PMID: 34398990

[ref5] Chen S. (2020). Parental investment after the birth of a sibling: the effect of family size in low-fertility China. Demography 57, 2085–2111. doi: 10.1007/s13524-020-00931-2, PMID: 33123983 PMC7736524

[ref6] Cheng T. O. (2005). One-child policy and increased mechanization are additional risk factors for increased coronary artery disease in modern China. Int. J. Cardiol. 100:333. doi: 10.1016/j.ijcard.2004.09.020, PMID: 15823644

[ref7] Cheng T. O. (2013). China’s little emperors: medical consequences of China’s one-child policy. Int. J. Cardiol. 168, 5121–5125. doi: 10.1016/j.ijcard.2013.08.074, PMID: 24071388

[ref9] DeGarmo D. S. Forgatch M. S. Martinez C. R. (1999). Parenting of divorced mothers as a link between social status and boys' academic outcomes: unpacking the effects of socioeconomic status. Child Dev. 70, 1231–1245. doi: 10.1111/1467-8624.00089, PMID: 10546342

[ref10] Ding Q. J. Hesketh T. (2006). Family size, fertility preferences, and sex ratio in China in the era of the one child family policy: results from national family planning and reproductive health survey. Br. Med. J. 333, 371–373. doi: 10.1136/bmj.38775.672662.80 16690642 PMC1550484

[ref11] Downey D. B. (2001). Number of siblings and intellectual development. The resource dilution explanation. Am. Psychol. 56, 497–504. doi: 10.1037//0003-066x.56.6-7.497, PMID: 11413873

[ref12] Draper P. Hames R. (2000). Birth order, sibling investment, and fertility among Ju/Hoansi (!Kung). Hum. Nat. 11, 117–156. doi: 10.1007/s12110-000-1016-0, PMID: 26193364

[ref13] Du W. Ke L. Wang Y. Hua J. Duan W. Barnett A. L. (2020). The prenatal, postnatal, neonatal, and family environmental risk factors for developmental coordination disorder: a study with a national representative sample. Res. Dev. Disabil. 104:103699. doi: 10.1016/j.ridd.2020.103699, PMID: 32623045

[ref14] Gillespie D. O. Russell A. F. Lummaa V. (2008). When fecundity does not equal fitness: evidence of an offspring quantity versus quality trade-off in pre-industrial humans. Proc. Biol. Sci. 275, 713–722. doi: 10.1098/rspb.2007.1000, PMID: 18211874 PMC2366115

[ref15] Hesketh T. Qu J. D. Tomkins A. (2003). Health effects of family size: cross sectional survey in Chinese adolescents. Arch. Dis. Child. 88, 467–471. doi: 10.1136/adc.88.6.467, PMID: 12765907 PMC1763126

[ref16] Hou X. H. Gong Z. Q. Wang L. J. Zhou Y. Su Y. (2020). A reciprocal and dynamic development model for the effects of siblings on Children's theory of mind. Front. Psychol. 11:554023. doi: 10.3389/fpsyg.2020.554023, PMID: 33192805 PMC7649281

[ref17] Hua J. Zhu L. Du W. Du L. Luo T. Wu Z. (2016). Infant’s sex, birth control policy and postpartum well-being: a prospective cohort study in Shanghai, China. BMJ Open 6:e012207. doi: 10.1136/bmjopen-2016-012207, PMID: 27855096 PMC5073912

[ref18] Huang Y. Tang W. Mu Y. Li X. Liu Z. Wang Y.. (2016). The sex ratio at birth for 5,338,853 deliveries in China from 2012 to 2015: a facility-based study. PLoS One 11:e0167575. doi: 10.1371/journal.pone.0167575, PMID: 27941978 PMC5152891

[ref19] Jaeger M. M. (2009). Sibship size and educational attainment. A joint test of the confluence model and the resource dilution hypothesis. Res. Soc. Stratif. Mobil. 27, 1–12. doi: 10.1016/j.rssm.2009.01.002, PMID: 22468016 PMC3314331

[ref20] Jamison C. S. Cornell L. L. Jamison P. L. Nakazato H. (2002). Are all grandmothers equal? A review and a preliminary test of the “grandmother hypothesis” in Tokugawa Japan. Am. J. Phys. Anthropol. 119, 67–76. doi: 10.1002/ajpa.10070, PMID: 12209574

[ref21] Kalmijn M. van de Werfhorst H. G. (2016). Sibship size and gendered resource dilution in different societal contexts. PLoS One 11:e0160953. doi: 10.1371/journal.pone.0160953, PMID: 27560371 PMC4999277

[ref22] Li H. Zhang J. Zhu Y. (2008). The quantity-quality trade-off of children in a developing country: identification using Chinese twins. Demography 45, 223–243. doi: 10.1353/dem.2008.0006, PMID: 18390301 PMC2831373

[ref23] Liu J. Duan C. Lummaa V. (2017). Parent-offspring conflict over family size in current China. American journal of human biology: the official journal of the Human Biology Council 29:e22946. doi: 10.1002/ajhb.22946, PMID: 28054420

[ref24] Park H. (2008). Public policy and the effect of sibship size on educational achievement: a comparative study of 20 countries. Soc. Sci. Res. 37, 874–887. doi: 10.1016/j.ssresearch.2008.03.002

[ref25] Pennington R. Harpending H. (1988). Fitness and fertility among Kalahari !Kung. Am. J. Phys. Anthropol. 77, 303–319. doi: 10.1002/ajpa.1330770304, PMID: 3228170

[ref26] R Core Team. (2021). *R: A Language and Environment for Statistical Computing*. Vienna, Austria: R Foundation for Statistical Computing.

[ref27] Ronfani L. Vecchi Brumatti L. Mariuz M. Tognin V. Bin M. Ferluga V.. (2015). The complex interaction between home environment, socioeconomic status, maternal IQ and early child neurocognitive development: a multivariate analysis of data collected in a newborn cohort study. PLoS One 10:e0127052. doi: 10.1371/journal.pone.0127052, PMID: 25996934 PMC4440732

[ref28] Schady N. (2011). Parents’ education, mothers’ vocabulary, and cognitive development in early childhood: longitudinal evidence from Ecuador. Am. J. Public Health 101, 2299–2307. doi: 10.2105/AJPH.2011.300253, PMID: 22021308 PMC3222428

[ref29] Shonkoff J. P. Richter L. van der Gaag J. Bhutta Z. A.. (2012). An integrated scientific framework for child survival and early childhood development. Pediatrics 129, E460–E472. doi: 10.1542/peds.2011-0366 22218840

[ref30] Shrout P. E. Bolger N. (2002). Mediation in experimental and nonexperimental studies: new procedures and recommendations. Psychol. Methods 7, 422–445. doi: 10.1037/1082-989X.7.4.422, PMID: 12530702

[ref31] Steelman L. C. (1985). A tale of 2 variables-a review of the intellectual consequences of Sibship size and birth-order. Rev. Educ. Res. 55, 353–386. doi: 10.3102/00346543055003353

[ref32] Tada Y. Keiwkarnka B. Pancharuniti N. Chamroonsawasdi K.. (2002). Nutritional status of the preschool children of the Klong Toey slum, Bangkok. Southeast Asian J. Trop. Med. Public Health 33, 628–637. PMID: 12693602

[ref33] Takeuchi H. Taki Y. Asano K. Asano M. Sassa Y. Yokota S.. (2021). Childhood socioeconomic status is associated with psychometric intelligence and microstructural brain development. Commun. Biol. 4:470. doi: 10.1038/s42003-021-01974-w 33927305 PMC8084976

[ref34] Teng X. Shane M. I. Pan S. (2020). The changing situation about maternal age, risk factors and pregnancy outcomes after the two-child policy: a retrospective cohort study. Ann. Palliat. Med. 9, 824–834. doi: 10.21037/apm.2020.04.27, PMID: 32312075

[ref35] Tong Y. Gong Q. (2020). The impact of child births on female labor force participation in China. China Popul. Dev. Stud. 3, 237–251. doi: 10.1007/s42379-019-00041-6, PMID: 31539074

[ref36] Tong S. Baghurst P. Vimpani G. McMichael A. (2007). Socioeconomic position, maternal IQ, home environment, and cognitive development. J. Pediatr. 151, 284–288.e1. doi: 10.1016/j.jpeds.2007.03.020, PMID: 17719939

[ref37] Ursache A. Noble K. G. (2016). Neurocognitive development in socioeconomic context: multiple mechanisms and implications for measuring socioeconomic status. Psychophysiology 53, 71–82. doi: 10.1111/psyp.12547, PMID: 26681619 PMC4685721

[ref38] Vukojević M. Zovko A. Talić I. Tanović M. Rešić B. Vrdoljak I.. (2017). Parental socioeconomic status as a predictor of physical and mental health outcomes in children-literature review. Acta Clin. Croat. 56, 742–748. doi: 10.20471/acc.2017.56.04.23, PMID: 29590731

[ref39] Wei M. Bian X. Squires J. Yao G. Wang X. Xie H.. (2015). Studies of the norm and psychometrical properties of the ages and stages questionnaires, third edition, with a Chinese national sample. Zhonghua Er Ke Za Zhi 53, 913–918. PMID: 26887546

[ref40] Whiteman S. D. Buchanan C. M. (2002). Mothers’ and children’s expectations for adolescence: the impact of perceptions of an older sibling’s experience. J. Fam. Psychol. 16, 157–171. doi: 10.1037/0893-3200.16.2.157, PMID: 12085729

[ref8] WHO Expert Consultation (2004). Appropriate body-mass index for Asian populations and its implications for policy and intervention strategies. Lancet 363, 157–163. doi: 10.1016/S0140-6736(03)15268-3 14726171

[ref41] Wu Q. (2015). Sibship size and Children’s family resources: findings from a nationally representative survey in China. J. Early Adolesc. 36, 575–594. doi: 10.1177/0272431615574885

[ref42] Wu S. Zhang D. Li X. Zhao J. Sun X. Shi L.. (2022). Siblings and early childhood development: evidence from a population-based cohort in preschoolers from Shanghai. Int. J. Environ. Res. Public Health 19. doi: 10.3390/ijerph192114559, PMID: 35565134 PMC9099463

[ref43] Zajonc R. B. Markus G. B. (1975). Birth-order and intellectual-development. Psychol. Rev. 82, 74–88. doi: 10.1037/h0076229, PMID: 27238555

[ref44] Zhong J. Gao J. Liu C. Huang J. Luo R. (2019). Quantity-quality trade-off and early childhood development in rural family: evidence from China’s Guizhou Province. Int. J. Environ. Res. Public Health 16. doi: 10.3390/ijerph16071307, PMID: 30979059 PMC6480094

[ref45] Zhou H. Mo D. Luo R. Yue A. Rozelle S. (2016). Are children with siblings really more vulnerable than only children in health, cognition and non-cognitive outcomes? Evidence from a multi-province dataset in China. Chin. World. Econ. 24, 3–17. doi: 10.1111/cwe.12155

[ref46] Zwicker J. G. (2021). Association of Gestational age and Developmental Coordination Disorder. JAMA Netw. Open 4:e2137599. doi: 10.1001/jamanetworkopen.2021.37599, PMID: 34905014

